# Combining hypothesis- and data-driven neuroscience modeling in FAIR workflows

**DOI:** 10.7554/eLife.69013

**Published:** 2022-07-06

**Authors:** Olivia Eriksson, Upinder Singh Bhalla, Kim T Blackwell, Sharon M Crook, Daniel Keller, Andrei Kramer, Marja-Leena Linne, Ausra Saudargienė, Rebecca C Wade, Jeanette Hellgren Kotaleski

**Affiliations:** 1 Science for Life Laboratory, School of Electrical Engineering and Computer Science, KTH Royal Institute of Technology Stockholm Sweden; 2 National Center for Biological Sciences, Tata Institute of Fundamental Research Bangalore India; 3 Department of Bioengineering, Volgenau School of Engineering, George Mason University Fairfax United States; 4 School of Mathematical and Statistical Sciences, Arizona State University Tempe United States; 5 Blue Brain Project, École Polytechnique Fédérale de Lausanne Lausanne Switzerland; 6 Department of Neuroscience, Karolinska Institute Stockholm Sweden; 7 Faculty of Medicine and Health Technology, Tampere University Tampere Finland; 8 Neuroscience Institute, Lithuanian University of Health Sciences Kaunas Lithuania; 9 Department of Informatics, Vytautas Magnus University Kaunas Lithuania; 10 Molecular and Cellular Modeling Group, Heidelberg Institute for Theoretical Studies (HITS) Heidelberg Germany; 11 Center for Molecular Biology (ZMBH), ZMBH-DKFZ Alliance, University of Heidelberg Heidelberg Germany; 12 Interdisciplinary Center for Scientific Computing (IWR), Heidelberg University Heidelberg Germany; Emory University United States; Emory University United States

**Keywords:** FAIR, modeling workflows, parameter estimation, mathematical modeling, uncertainty quantification, synaptic plasticity

## Abstract

Modeling in neuroscience occurs at the intersection of different points of view and approaches. Typically, hypothesis-driven modeling brings a question into focus so that a model is constructed to investigate a specific hypothesis about how the system works or why certain phenomena are observed. Data-driven modeling, on the other hand, follows a more unbiased approach, with model construction informed by the computationally intensive use of data. At the same time, researchers employ models at different biological scales and at different levels of abstraction. Combining these models while validating them against experimental data increases understanding of the multiscale brain. However, a lack of interoperability, transparency, and reusability of both models and the workflows used to construct them creates barriers for the integration of models representing different biological scales and built using different modeling philosophies. We argue that the same imperatives that drive resources and policy for data – such as the FAIR (Findable, Accessible, Interoperable, Reusable) principles – also support the integration of different modeling approaches. The FAIR principles require that data be shared in formats that are Findable, Accessible, Interoperable, and Reusable. Applying these principles to models and modeling workflows, as well as the data used to constrain and validate them, would allow researchers to find, reuse, question, validate, and extend published models, regardless of whether they are implemented phenomenologically or mechanistically, as a few equations or as a multiscale, hierarchical system. To illustrate these ideas, we use a classical synaptic plasticity model, the Bienenstock–Cooper–Munro rule, as an example due to its long history, different levels of abstraction, and implementation at many scales.

## Introduction

**Figure 1.**
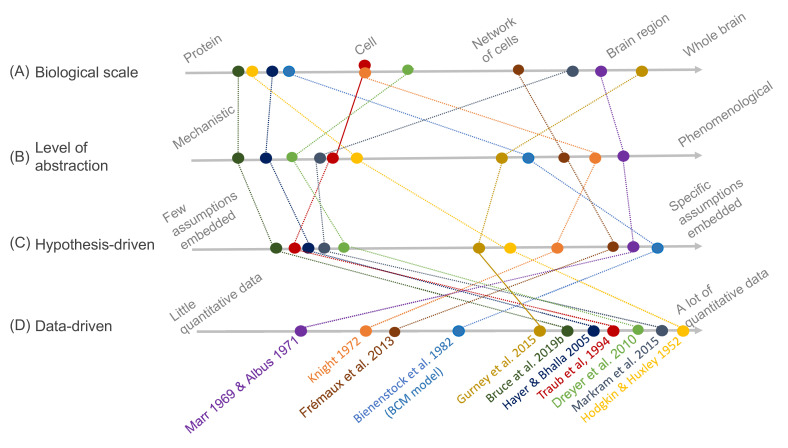
Schematic illustration of different types of models and their relative relationships. A subset of neuroscience models are visualized based on four criteria: (**A**) *biological scale*, (**B**) *level of abstraction*, (**C**) the degree to which a *hypothesis-driven*, or (**D**) *data-driven* approach have been used for the model construction. Illustration includes examples from the text as well as several classical models: Albus, 1971 (hypothesis-driven phenomenological model of the cerebellar circuitry as a pattern recognition system); Bienenstock et al., 1982 (algebraic bidirectional synaptic plasticity rule); Bruce et al., 2019b (mechanistic molecular model of the regulation of adenylyl cyclase 5 by G-proteins in corticostriatal synaptic plasticity induction); Dreyer et al., 2010 (data-driven mechanistic model of tonic and phasic dopamine release and activation of receptors in the dorsal striatum), Frémaux et al., 2013 (hypothesis-driven phenomenological model of a reward-modulated spike-timing-dependent learning rule for an actor-critic network); Gurney et al., 2015 (basal ganglia model with corticostriatal reinforcement learning using data-driven synaptic plasticity rules); Hayer and Bhalla, 2005 (data-driven biochemical bidirectional synaptic plasticity model involving calcium/calmodulin-dependent protein kinase II [CaMKII]); Hodgkin and Huxley, 1952 (classic biophysical model of action potentials); Knight, 1972 (hypothesis-driven phenomenological model of stimulus encoding into a neuronal population); Markram et al., 2015 (large-scale digital reconstruction of somatosensory cortex microcircuitry); Marr, 1969 (cerebellar algorithm model); Traub et al., 1994 (biophysical hippocampal CA3 neuron model showing the importance of dendritic ion channels).

**Figure 2.**
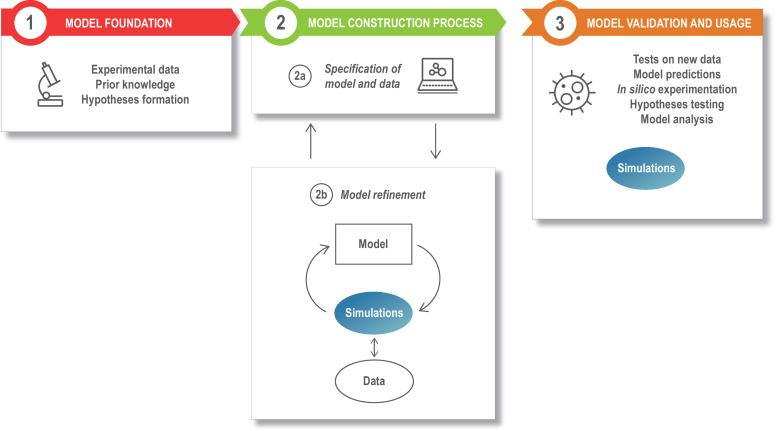
The modeling process. Model development starts with assembling the information that is the foundation of the modeling study, such as the relevant experimental literature, published models, and additional experimental results (box 1). This is specified in a structured model and data format (box 2a), and the model is next refined and updated (box 2b). The model refinement step is an iterative process, where the model is simulated a large number of times to update the model parameters so that the model captures specific experimental data (quantitatively) or phenomena (qualitatively). Once developed, the model can be exploited for a variety of applications (box 3).

Dynamical models provide an essential counterpart to experiments in the endeavor to understand the brain, and today a large ecosystem of model types and approaches exists (Figure 1). For example, hypothesis-driven modeling typically brings a question into focus so that a model is constructed to investigate a specific hypothesis about how the brain works. Data-driven modeling, on the other hand, often follows a more unbiased approach, with model construction informed by the computationally intensive use of data. Although hypothesis- and data-driven modeling approaches are not mutually exclusive, a plethora of modeling formalisms, simulation platforms, and data formats has fragmented the neuroscience modeling community, particularly when it comes to modeling at different biological scales or levels of abstraction. This diversity is beneficial: most models and model building tools have a clear and specific role, but at the same time combining these approaches in an interoperable way would have an immense impact on our understanding of the brain. The FAIR (Findable, Accessible, Interoperable, Reusable) principles (Wilkinson et al., 2016) have been widely discussed and implemented for data management (Wittig et al., 2017), and recently also for computational workflows (Goble et al., 2020). We suggest that these principles would be beneficial for models and modeling workflows within neuroscience as well. In this review, we examine the different steps of a general modeling workflow and look at different alternatives for model building, refinement, analysis, and use (Figure 2). For each step we consider how the FAIR principles can be applied to the different aspects of the modeling process. We focus on models at the intracellular or cellular level, where the field of computational neuroscience meets systems biology modeling, with an emphasis on models of brain plasticity and learning. However, the discussed methodology and concepts can be applied to other biological systems and scales as well (see, e.g., Einevoll et al., 2019).

As an illustration of the wide variety of model types, Figure 1 depicts a number of models arranged according to four criteria: *biological scale*, *level of abstraction*, and to what degree *hypothesis-* and *data-driven* approaches were used during model development. The axes of the figure are qualitative, additional dimensions could be considered, and the precise arrangement of the models could be interpreted differently as there are no strict metric spaces defined. Regarding *biological scale*, we note that experimental data can be measured and applied at different resolutions — from a molecular signaling pathway in a single synapse to an entire brain. This is reflected in the models, which exist, for example, from the level of molecular interactions important for plasticity in specific synapses (Bruce et al., 2019b) to brain structures with an aim of understanding generalized learning rules (Gurney et al., 2015). Because of different modeling goals, models are also constructed with different *levels of abstraction*. For example, the dopamine signal is important for reward learning, addiction, and motivation, and has been modeled phenomenologically with a reward-prediction error (Frémaux et al., 2013) or more mechanistically, taking into account dopamine diffusion and the affinity of dopamine receptors (Dreyer et al., 2010). Figure 1 also includes the *data-* and *hypothesis-driven* aspects of models. Models that have a high score on the data axis are typically based on a large amount of quantitative data and/or have been developed using data science methodology. Models that have a high score on the hypothesis axis, on the other hand, are strongly driven by a particular question or idea, and one aim could be to investigate if some assumed mechanism explains the studied phenomena. As a side note, we have used the concepts of ‘top-down’ and ‘bottom-up’ modeling restrictively within this article since those concepts are often implicitly linked to different field-dependent contexts, especially concerning the intensive use of data in systems biology and neuroscience, for example compare (Bruggeman and Westerhoff, 2007) and (Eliasmith and Trujillo, 2014; Dudai and Evers, 2014).

All these different types of models can be useful, but their value is greatly increased if they can be integrated to understand the same experimental data and phenomena at multiple scales or different levels of abstraction. For example, an abstract model of reward prediction can be given details through multicompartmental modeling, allowing this new form to be directly compared to experimental data. An experimentally validated model can then be used for designing new experiments or to predict efficient therapeutic interventions. Across all model types, it is also important to support new tools for model validation and refinement including methods for efficient parameter estimation, global sensitivity analysis, uncertainty quantification, and other data science approaches (Alber et al., 2019). Not only is this critical for increasing the accuracy and reproducibility of model development, but also for the efficient inclusion of new data. A FAIR modeling infrastructure that includes both models, data and software is essential for establishing synergy among modeling approaches and for the refinement and validation of models.

Wilkinson et al., 2016 subdivided the FAIR principles into 15 subcategories that describe important details regarding scientific data that are Findable, Accessible, Interoperable, and Reusable. The intent was to not only consider ‘data’ in the conventional sense but also the algorithms, tools, and workflows that led to the data. In this review, we follow this path and consider FAIR with regard to models and modeling tools in addition to experimental data. While all 15 subcategories should be considered by modeling software and database developers, here we focus on the subset of actions that can be taken by individual researchers, and we avoid technological complexity in our suggestions. The principles of *findability* and *accessibility* for models and modeling workflows both require the availability of databases and repositories where models, software and associated data can be stored, and the use of persistent, unique identifiers and metadata. When it comes to *interoperability* of models, our main focus concerns the ability to run the same model using different simulation platforms and model analysis tools. Nonetheless, we also discuss interoperability across neighboring scales, such as when the output from a model at a finer resolution is used as the input to a model at a more coarse-grained resolution. Concerning *reusability*, our main focus is that different laboratories should be able to reuse and rebuild each other’s models efficiently, while acknowledging the provenance of a model, as well as the data used to constrain and validate the model.

Reusability is related to the issue of research reproducibility, which has been discussed within many scientific fields. In a recent study (Tiwari et al., 2021), the authors attempted to reproduce more than 400 kinetic models of biological processes published in peer-reviewed research articles in conjunction with the curation process in the BioModels (Chelliah et al., 2015) repository, and they found that only about half of the models could be directly reproduced. They note that there is a difference between reproducibility and repeatability. In principle, a modeling process is repeatable if someone else can run the same code and get the same results. Reproducibility on the other hand requires that a different researcher starting from the same information but with another implementation reaches the same result. Reproducibility therefore provides a stronger quality check for computational scientific results. Other interpretations of these terms exist, as discussed in a recent study (Plesser, 2017), but here we follow the terminology described above.

In order to further develop modeling workflow capabilities fulfilling the FAIR criteria, a tightly integrated ecosystem of databases, software, and standardized formats is needed. Many of these pieces exist, supporting the modeling process from initial model development, to the use of models in exploration and prediction. For example, over the years, the field of computational neuroscience has developed a set of *open-source simulation environments* (Gewaltig and Cannon, 2014). At the same time, the community is depositing published models into *online repositories*, such as model databases (Birgiolas et al., 2015; Gleeson et al., 2019; Hines et al., 2004; Li et al., 2010) and GitHub (github.com), for others to inspect or use. Simulator independent, *standardized model specifications* such as the Systems Biology Markup Language (SBML) (Hucka et al., 2003) and NeuroML (Gleeson et al., 2010) have been developed to promote model interoperability and reproducibility. Finally, increasingly, *experimental data* are being used extensively in the modeling process, and frameworks for model calibration (Ashyraliyev et al., 2009; Mitra and Hlavacek, 2019; Sun et al., 2012) and for validating models against data (Omar et al., 2014) are available, or under development. What remains is to develop standardized workflows and data formats that integrate the components of this ecosystem to facilitate reusing, extending, and comparing models, within and across scales. The aim is to promote interoperability among different software packages used within neuroscience, to increase shareability of models, and at the same time improve transparency and reproducibility with regard to the model building process and the data used for parameter calibration and validation. All this can in the end also contribute to increased automation of the entire process to set the stage for wider use of data intensive methods.

As we consider the modeling workflow shown in Figure 2, we discuss how FAIR principles can be introduced to this process. To illustrate these steps with a concrete example, we use a classical model of activity-dependent synaptic plasticity, the Bienenstock–Cooper–Munro (BCM) rule (Bienenstock et al., 1982), described in detail below. Here, plasticity depends on pre- and postsynaptic activity, and leads to long-term depression (LTD) or long-term potentiation (LTP). The BCM rule is chosen as an example because it was first modeled phenomenologically and also has been subsequently reproduced using mechanistic models with increasing levels of detail as more data and knowledge have accumulated (Lisman, 1989). More recent models of signaling underlying the BCM rule are substantially data driven (Castellani et al., 2005; Hayer and Bhalla, 2005).

### An example: the BCM rule

The BCM rule is a synaptic plasticity model (Bienenstock et al., 1982) formulated in the context of the development of orientation selectivity in the visual system. It states that as postsynaptic activity increases, there are two domains of plasticity that induce either depression or potentiation. This rule has since been used as the basis for considerable theoretical and experimental work on synaptic plasticity, and here it is employed to illustrate approaches for modeling plasticity and to motivate our discussion of modeling workflows and FAIR approaches.

**Figure 3.**
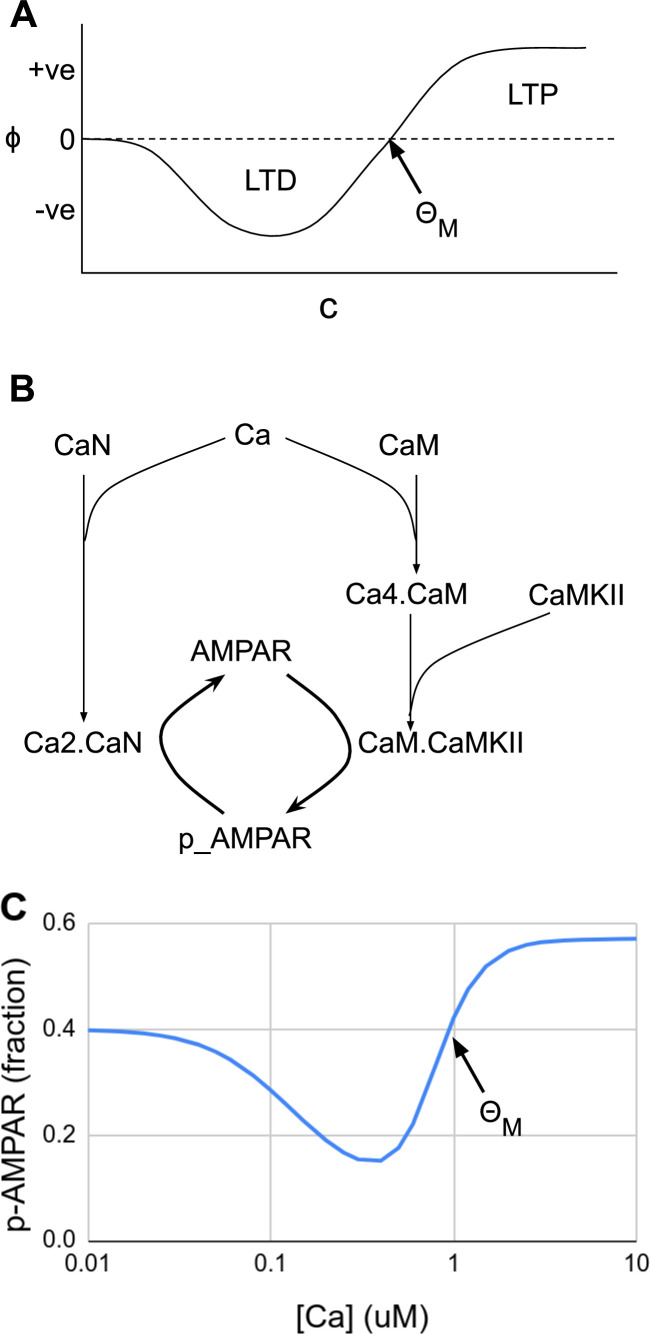
Abstract and mechanistic versions of the Bienenstock–Cooper–Munro (BCM) rule model. (**A**) Original version where the rate of plasticity change, ϕ, is a function of the stimulus strength c. At the threshold $\Theta_{M}$ the sign of synaptic change flips from negative to positive. (**B**) Simplified mechanistic (chemical) model based on known pathways that could implement the BCM rule. The calcium stimulus activates both a kinase, CaMKII, and a phosphatase, CaN. These act in an antagonistic manner on the AMPA receptor, leading to its dephosphorylation and removal from the synapse when the calcium concentration, [Ca^2+^], is moderate, but insertion when [Ca^2+^] is high. The output from the model is p_AMPAR, the phospho-form of AMPAR, which is inserted into the membrane. (**C**) Simulated response of the model in Panel B, measured as phosphorylated receptor p_AMPAR. This curve has the same shape as the abstract BCM curve model in Panel A, including a threshold level of [Ca^2+^] = $\Theta_{M}$ at which the synaptic change as measured by AMPA phosphorylation changes sign from negative to positive. The basal fraction of p_AMPAR is 0.4. Model from accession 96 on DOQCS (see Table 1, Row 15).

The original BCM rule describes the rate of change of synaptic weight as

$$\frac{dm\left(t\right)}{dt}=\phi \left(c\left(t\right)\right)\;s\left(t\right)\;-\;\epsilon \;m\left(t\right)\;,$$

where is the synapse input current, ϵ is the time constant of synaptic decay, and c is the postsynaptic activity. ϕ represents the postsynaptic activation function, formulated as

$$\phi (c)<0\;for\;c<\Theta_{M}\;and\;\phi (c)>0\;for\;c>\Theta_{M}$$

where $\Theta_{M}$ is the activity threshold at which the synaptic strengths are modified. The BCM rule is phenomenological because ϕ does not map to any biological mechanisms, and the authors showed that this learning rule can account for the formation of orientation selectivity for a wide range of values for $\Theta_{M}$_._ A typical graph of ϕ is depicted in Figure 3A, and similar curves have been obtained experimentally (Kirkwood et al., 1996).

This model was published in a widely read society journal and predates today’s FAIR principles. The article included simulations of the model equations, but, lacking today’s open-source, code-sharing infrastructure, readers were left to their own skills to replicate these implementations. Like many abstract models, the BCM model is a mostly qualitative representation of concepts derived from a substantial amount of data, including many features of synaptic plasticity, network-level data for orientation selectivity, and data for the degradation of orientation selectivity in the case of monocular deprivation and similar experiments. The publication includes simulations of several such cases, showing a qualitative match to data but does not explain how parameters were derived. In summary, due to the minimal, abstract nature of the model, the BCM study is not difficult to reproduce; however, there is no reference implementation available for simulations, and the parameters for several simulations are not available. Thus, the published simulations and parameters are not consistent with today’s FAIR principles.

Moving forward a few decades, one finds mechanistic, mass-action models of synaptic signaling and the BCM rule, where the relevance of FAIR and data-driven approaches becomes more apparent. First, the quantities under consideration (input activity and synaptic strength) correspond directly to synaptic molecules. Experimental data tell us that intracellular calcium concentration is a good proxy for postsynaptic activity, c, and that the phosphorylation state of the AMPA receptor is one of the proxies for synaptic efficacy, m. Filling in the reaction diagram, one obtains a simplified reaction network of the form shown in Figure 3B. Remarkably, the properties of these molecules result in a response that resembles the BCM curve. The phosphatase calcineurin (CaN) has a high affinity for calcium bound calmodulin, and also can partly become activated directly by calcium (Creamer, 2020). Hence, it is activated at moderate calcium and triggers an initial decrease in phosphorylated AMPAR and LTD (ϕ(c)<0 from the BCM formulation). Calcium/calmodulin-dependent protein kinase II (CaMKII) has a weaker affinity for calcium bound calmodulin, but is present in overwhelming amounts, so its activation surpasses CaN at high calcium concentrations, thus implementing ϕ(c)>0 for $c>\Theta_{M}$, and leading to increase in phosphorylated AMPAR and thus LTP. The resulting curve from this mechanistic but simplified model matches the BCM curve (Figure 3C). More detailed mechanistic versions of the BCM model have been implemented using known synaptic chemistry in considerably greater detail (Lisman, 1989; Hayer and Bhalla, 2005).

How FAIR and data driven are these more recent models? The Lisman, 2017 is not consistent with our current definition of FAIR: it provides full disclosure of equations and rate constants, but reuse requires reimplementation. The more recent model (Hayer and Bhalla, 2005) exists in at least two open-access databases, the Database of Quantitative Cellular Signaling, DOQCS (Sivakumaran et al., 2003), and BioModels (Li et al., 2010), hence is findable and accessible. It has been converted to the standardized SBML model description format, promoting interoperability and reusability. The DOQCS version has citations and calculations for the derivation of some of the parameters, and the BioModels version maintains the provenance, referencing the DOQCS entry from which it was derived.

Parameter access greatly improves as models become FAIR. For example, Lisman’s study acknowledges the original BCM model as a key motivation (Lisman, 1989), but no parameters were available, leading Lisman to use ‘plausible’ estimates for rates that form the BCM curve. In contrast, Hayer and Bhalla had access to reaction schemes and rates from databases such as DOQCS (Sivakumaran et al., 2003), and model construction was facilitated considerably by the FAIR principles. However, the database version of this model does not include the provenance for the data underlying some of the parameters, in part because data were unavailable. Building on Hayer and Bhalla, 2005, numerous studies benefited from increasing convergence toward FAIR principles and have derived model structures and parameters from models placed in accessible repositories (e.g., Li et al., 2012; Nakano et al., 2010; Mäki-Marttunen et al., 2020).

To summarize, the history of models that implement the BCM rule demonstrates a clear progression from abstract, qualitative, and unFAIR formulations to more mechanistic, quantitative, and FAIR formulations of the same conceptual model using data-driven parameterization. This progression is a natural consequence of the accumulation of experimental data and nascent concepts of FAIR principles. In what follows, we consider how to improve data-driven models further through FAIR workflows and data science-based approaches.

## Framework for FAIR modeling workflows

A typical model development process can be divided into different stages or modules, as illustrated in Figure 2. The *model foundation* (Figure 2, box 1) stage corresponds to the process of collecting the experimental data and prior knowledge that will be used for the rest of the modeling process and combine these into an initial plan for the *model structure* (or topology), that is, a description of the different model entities and how they interact. In the next stage, the *specification of the model and data* (Figure 2, box 2a), the information from step 1 is formalized into a standardized, machine readable format. During the *model refinement* stage (Figure 2, box 2b), the model is transferred to a mathematical formalism, simulated, calibrated, and refined. After model refinement is complete, the specification files are to be updated with this new information. Finally, during the *model usage* stage (Figure 2, box 3), the resulting model is validated, analyzed, and used to predict or investigate additional phenomena. Some examples of tools and formats that can be used in this process are given in Table 1–4. Note that these tables do not provide an exhaustive list, but rather a starting point for discussion.

### Model foundation: experimental data and prior knowledge

The *Model foundation* (Figure 2, box 1) may include information from published literature, novel unpublished experimental data, experimental data and models retrieved from databases, or, for multiscale modeling, information from models at other biological scales (Table 1). These different data sources may include qualitative as well as quantitative data. Qualitative data give information about qualitative traits such as the possibility that two proteins interact or knowledge of positive or negative correlations, which are useful for defining the structure of the model, that is the modeling entities and how they interact. As an example, in the case of intracellular pathway models, such model structure defines which molecular species and reactions to consider, as exemplified in the chemical signaling network in Figure 3B. Quantitative data, on the other hand, are needed to specify quantitative entities in models, such as rate constants, equilibrium constants, or initial concentrations of molecules. This can be done either through direct measurements or indirectly during the model refinement stage (Figure 2, box 2b), where model outputs are compared to experimental readouts.

**Table 1.** Databases in cellular neuroscience and systems biology. These are some of the commonly used databases for creating and constraining models at the intracellular and cellular scale.

	Database, alphabetically	Purpose/focus	Reference	Homepage
	*Computational models*			
1	BioModels Database	Physiologically and pharmaceutically relevant mechanistic models in standard formats	Li et al., 2010	http://www.ebi.ac.uk/biomodels/
2	MoDEL Central Nervous System	Atomistic-MD trajectories for relevant signal transduction proteins		http://mmb.irbbarcelona.org/MoDEL-CNS/
3	NeuroML-DB	Models of channels, cells, circuits, and their properties and behavior	Birgiolas et al., 2015	https://neuroml-db.org/
4	NeuroElectro	Extract and compile from literature electrophysiological properties of diverse neuron types	Tripathy et al., 2014	https://neuroelectro.org
5	ModelDB	Computational neuroscience model	McDougal et al., 2017	https://senselab.med.yale.edu/modeldb/
6	Ion Channel Genealogy	Ion channel models	Podlaski et al., 2017	https://icg.neurotheory.ox.ac.uk/
	*Experimental data*			
7	Allen Brain Atlas	Human and mouse brain data	Lein et al., 2007	http://www.brain-map.org/
8	BRENDA	Enzyme kinetic data	Chang et al., 2021	https://www.brenda-enzymes.org/
9	CRCNS - Collaborative Research in Computational Neuroscience	Forum for sharing tools and data for testing computational models and new analysis methods	Teeters et al., 2008	https://CRCNS.org
10	NeuroMorpho	Neuronal cell 3D reconstructions	Ascoli et al., 2007	http://neuromorpho.org/
11	Protein Data Bank (PDB)	3D structures of proteins, nucleic acids, and complex assemblies	wwPDB consortium, 2019	http://www.wwpdb.org/
12	Sabio-RK	Curated database on biochemical reactions, kinetic rate equations with parameters and experimental conditions	Wittig et al., 2012	http://sabio.h-its.org/
13	Yale Protein Expression Database (YPED)	Proteomic and small molecules	Colangelo et al., 2019	https://medicine.yale.edu/keck/nida/yped/
	*Experimental data and models*			
14	Channelpedia	Ion channel data and channel models	Ranjan et al., 2011	https://channelpedia.epfl.ch/
15	DOQCS The Database of Quantitative Cellular Signaling	Kinetic data for signaling molecules and interactions	Sivakumaran et al., 2003	http://doqcs.ncbs.res.in/
16	EBRAINS (including EBRAINS Knowledge Graph)	Digital research infrastructure that gathers data, models and tools for brain-related research		https://ebrains.eu (https://search.kg.ebrains.eu)
17	FAIRDOMHub	The FAIRDOMHub is a repository for publishing FAIR Data, Operating procedures and Models for the Systems Biology community	Wolstencroft et al., 2017	https://fairdomhub.org/
18	Open Source Brain	A resource for sharing and collaboratively developing computational models of neural systems	Gleeson et al., 2019	https://www.opensourcebrain.org/

Findable data, data provenance, and data reusability are key components of the FAIR principles (Wittig et al., 2017) and are important for this first step of the modeling process. FAIR modeling workflows are facilitated by the many databases that can be used to constrain neuroscience models, which also includes databases for models from earlier studies. As mentioned above, the focus of this review is on cellular- and intracellular-level models, and data and model sharing resources for this level can be found in Table 1. For example, summary statistics (e.g. [Tripathy et al., 2014]) and morphological data for different neuron types (e.g. [Ascoli et al., 2007]), and integrated data for gene expression, connectivity, and neuroanatomy (e.g. Allen Brain Atlas [Lein et al., 2007; Sunkin et al., 2013]) are useful for constructing models at the cellular level. Often, published computational neuroscience models are shared via ModelDB (McDougal et al., 2017), which includes over 1700 models and is tightly coupled with NeuronDB, a database of neuronal properties that are used to constrain models based on experimental observations. Other repositories of neuroscience models include NeuroML-DB with over 1500 models in NeuroML format at multiple scales (Birgiolas et al., 2015), and Open Source Brain, a platform for collaborating, simulating, and sharing neuroscience models (Gleeson et al., 2019). Systems biology databases, such as BRENDA (Chang et al., 2021) and SABIO-RK (Wittig et al., 2018), contain kinetic data on enzyme kinetics and protein–protein interactions, which are critical for modeling subcellular signaling pathways. SABIO-RK is a curated database that was specifically designed to facilitate systems biology modeling, which provides reaction kinetics data along with information on experimental conditions, units, and kinetic rate equations. Systems biology models are also available through model databases such as BioModels, which includes many models that are relevant to systems biology level models in neuroscience (Malik-Sheriff et al., 2020). One important feature of databases for experimental data and models is the accompanying metadata, which are critical for finding and using these data. For example, associating ontology references to biological terms makes it possible to use the associated domain knowledge in formulating search strategies (Birgiolas et al., 2015) and in the model building process.

For the synaptic plasticity model example described here, the models that employ the BCM rule have progressively used more information and data from different databases as new models have been developed. As mentioned above, mechanistically detailed versions of the synaptic plasticity model use model reaction schemes and reaction rates from existing databases: DOQCS (accessions 59–64) and the BioModels Database. Additionally, several subsequent cellular and network models available in ModelDB rely on various versions of the synaptic plasticity model (Jedlicka et al., 2015; Wilmes and Clopath, 2019; Mäki-Marttunen et al., 2020; Manninen et al., 2020).

In spite of the large amounts of data available at different biological scales, data are still sparse if one wants to build mechanistic, ‘bottom-up’ models to better understand multiscale, causal chains of events such as how properties of proteins affect cellular-level phenomena or how cellular and synaptic properties affect network dynamics and function (Klipp et al., 2010). However, it is sometimes possible to use predictions from a model at finer biological resolution to provide constraints to model parameters at the next level of abstraction (Boras et al., 2015; Stein et al., 2007; Wang et al., 2018; Xie et al., 2014). For example, molecular dynamics simulations which use biomolecular structural data can provide important quantitative or qualitative constraints on kinetic parameters, binding affinities, and their modulation by allosteric interactions in intracellular signaling pathway models (Bruce et al., 2019b, Bruce et al., 2019a; Gabdoulline et al., 2003; van Keulen et al., 2022). Tools exist to facilitate the use of biomolecular structural data in model building (Stein et al., 2007). For example, SYCAMORE (Weidemann et al., 2008) can use protein structural data along with published kinetic measurements for parameter assignment in the construction of signaling pathway models. Additional modeling tools and use cases for reuse of model components in multiscale models are being implemented through the EBRAINS infrastructure (ebrains.eu). This combination and reuse of model components at multiple biological scales emphasizes the importance of FAIR principles, since model components must be not only *reusable*, but also *interoperable* and *findible* for this to be achieved.

### Specifications of model and data

An important part of a FAIR modeling workflow is the specification of the model and experimental data (Figure 2, box 2a). Standardized formats for models have been developed with a goal of making it possible to efficiently reproduce the modeling results of another laboratory. Specifically, standardized model formats are critical for both interoperability and reuse. If we want to reproduce the entire modeling process, including model refinement, this requires information not only about the *model*, but also the *quantitative data* used for calibration and validation, a description of the different *simulation experiments* performed (corresponding to the biological experiments to be reproduced or predicted), as well as the *prior assumptions* made on parameters, all in machine readable format. A minimal requirement on such a specification is (but not limited to):

- the model
  ○ the model constituents, for example molecular species in a biochemical signaling model;
  ○ the interactions, for example reactions in a biochemical signaling model or synaptic connectivity in neuronal network model;
  ○ the parameters, for example synaptic conductances, reaction rates;
- the simulation experiments
  ○ the changes that are made to the model to replicate each of the biological experiments in for example initial conditions, parameters, or input functions;
- the quantitative experimental data
  ○ used to constrain model parameters;
  ○ used for validation – if model validation was performed;
  ○ the mapping between model output and experimental data readouts;
- the prior (i.e., before the model refinement) assumptions on parameter values;
- the posterior (i.e., after the model refinement) estimates for parameter values.

The prior assumptions on parameter values inform the model refinement process and may contain parameter ranges or distributions, or previously estimated values based on data, including error bounds. To be complete, the model and data specification may also include metadata on the calibration method used and identifiers for all model entities. This recommendation is similar to MIASE (Minimum Information About a Simulation Experiment) compliance; in Waltemath et al., 2011 the authors list more specific measures that authors can take to make their models easier to simulate by others. These simulation experiments can range from a time series simulation, to a parameter scan, to a sensitivity or bifurcation analysis, methods that are further described below. COMBINE (co.mbine.org, Hucka et al., 2015) is an initiative to coordinate the development of the various community standards and formats for computational models.

There are many ways to archive and share this information, and we discuss several possibilities. One approach is to maintain all information in one location, but often, a more distributed approach is required. In either case, the information should be clearly described and linked through metadata, including unique persistent identifiers to the different components like models or experimental data. In Table 2, we list various model description standards and file formats that can be used, and in later sections we describe ways to retrieve permanent unique identifiers.

**Table 2.** Model standards and file formats in cellular neuroscience and systems biology. The formats described in this table allow standardized representation of models and their porting across simulation platforms.

	Name	Purpose	Webpage	Reference
	*Formats for intracellular models in systems biology*			
1	SBML	Systems biology markup language, for storing and sharing models	https://sbml.org	Hucka et al., 2003
2	SBtab	Systems Biology tables, for storing models (and data for parameter estimation) in spreadsheet form	https://sbtab.net/	Lubitz et al., 2016
3	CellML	Store and exchange mathematical models, primarily in Biology	https://www.cellml.org/	Kohl et al., 2001
	*Formats for cellular and network-level models in Neuroscience*			
4	NeuroML	A XML-based description language that provides a common data format for defining and exchanging descriptions of neuronal cell and network models	http://www.neuroml.org/	Gleeson et al., 2010
5	NineML	Unambiguous description of neuronal network models	https://github.com/INCF/nineml-spec	Raikov et al., 2011
6	NestML	Domain-specific language for the specification of neuron models (python)	https://github.com/nest/nestml	Plotnikov et al., 2016
	*Custom formats for specific simulators*			
7	sbproj	Simbiology Project file	https://se.mathworks.com/products/simbiology.html	Schmidt and Jirstrand, 2006
8	COPASI project file	COPASI native format for models and simulations	http://copasi.org/	Hoops et al., 2006
9	SONATA	Efficient descriptions of large-scale neural neworks	https://github.com/AllenInstitute/sonata	Dai et al., 2020
10	JSON (HillTau)	JSON files for FindSim and HillTau model reduction method	https://github.com/BhallaLab/HillTau	Bhalla, 2020
11	MOD	Expanding NEURON’s repertoire of mechanisms with NMODL	https://www.neuron.yale.edu/neuron/	Hines and Carnevale, 2000
	*Formats for specification of parameter estimation problems*			
12	SBtab	Systems Biology tables, for storing both models and data for parameter estimation in spreadsheet form	https://sbtab.net/	Lubitz et al., 2016
13	PEtab	Interoperable specification of parameter estimation problems in systems biology	https://github.com/PEtab-dev/PEtab	Schmiester et al., 2021
	*Formats for specification of experiments and data*			
14	SED-ML	Simulation Experiment Description Markup Language	https://sed-ml.org	Waltemath et al., 2011

At the systems biology level, examples of existing storage formats for the information above include SBtab (Lubitz et al., 2016) and the JSON format used in FindSim (Viswan et al., 2018). SBtab is well structured, so it is machine readable as well as human readable, and it is able to capture information about biochemical network models and associated calibration data in a single location. SBtab has defined fields for sbo (systems biology ontology) terms (Courtot et al., 2011), as well as database identifiers (e.g. to UniprotKB [UniProt Consortium, 2021] or KEGG [Kanehisa and Goto, 2000]), and additional information can be included to map between experimental readouts and model simulation outputs. In the FindSim framework, an experiment is codified as a file with information about the experimental stimuli and the readouts, as well as how they map to the model output.

SBML provides a standardized machine readable markup language for sharing *only the model description* that is supported by many simulation platforms. For example, the MOOSE and STEPS simulators accept models in the SBML format. One advantage of SBML is that it describes all aspects of a model using a simulator-independent approach, but a disadvantage is that an SBML file can be hard to write by hand so that SBML files typically are created using an applications programming interface (API) such as libSBML. In some cases, a simulation platform may provide an API as the primary method for model specification. This API approach is taken by packages such as rxd (Newton et al., 2018). Another approach to model handling, taken by VFGEN (vector field generator, Weckesser, 2008), is independent of biology but specific to differential equations. VFGEN converts an input file that describes a differential equation to source code for many different solvers in various languages (e.g., cvode in C, javascript, gsl in C, RADAU5 in Fortran).

For neuron and circuit models at other scales, several standards have been developed and adopted to varying degrees for use by simulation platforms. Examples of these include NeuroML, CellML, SONATA, and others (see Table 2). In particular, NeuroML is an International Neuroinformatics Coordinating Facility (INCF) (Abrams et al., 2021) endorsed standard supported by dozens of downstream software packages. Well-tested software libraries can be used to convert NeuroML model description files to code for specific simulators such as NEURON or MOOSE or to programming languages such as Python or Matlab (Gleeson et al., 2010). NeuroML also includes defined fields for ontological metadata, such as the terminology provided by the Neuroscience Information Framework (NIF) ontologies (Bug et al., 2008). There are standards for data as well, which aid the comparison of model simulation outputs to experimental data. Neurodata Without Borders (NWB) (Teeters et al., 2015; Rübel et al., 2019) and neuroscience interchange format (NIX) (Stoewer et al., 2014) are both standards for describing electrophysiology data that are recognized by the INCF.

How should the abstract and the mechanistic BCM models be specified using a standardized approach? The abstract version of the model should be defined using an existing simulator independent standard, such as SBtab or SBML, and should include the published data for the shape of the curve (Kirkwood et al., 1996). Similarly, the mechanistic version of the BCM model should be specified using SBML or SBtab, where the chemical experiments are defined using, for example, SBtab or JSON for FindSim. An important validation step for the mechanistic model would be to recreate the same final curve used to constrain the abstract model, demonstrating that the two formulations converge. To be able to reproduce the parameter estimation process, information on prior assumptions made on possible parameter ranges should be added to the files, as well as metadata concerning the optimization procedure.

### Model simulation

Over the past 25 years, many *general purpose* and *domain-specific* simulation tools have been developed for modeling and simulation of biochemical and biophysical events in cells and circuits and even whole brain regions (Figure 2, boxes 2b and 3). Representative examples of these tools are provided in Table 3.

**Table 3.** Software for model simulation. These tools span a wide range of scales and levels of abstraction.

	Name	Purpose	Interchange file formats supported	Homepage	RRID	Reference
	*Molecular level*					
1	BioNetGen	Rule-based modeling framework (NFsim)	BNGL, SBML	http://bionetgen.org/		Harris et al., 2016
2	COPASI	Biochemical system simulator	SBML	http://copasi.org/	SCR_014260	Hoops et al., 2006
3	IQM Tools	Systems Biology modeling toolbox in MATLAB; successor to SBPOP	SBML	https://iqmtools.intiquan.com/		
4	MCell	Simulation tool for modeling the movements and reactions of molecules within and between cells by using spatially realistic 3D cellular models and specialized Monte Carlo algorithms	SBML	https://mcell.org/	SCR_007307	Stiles et al., 1996; Stiles and Bartol, 2001, Kerr et al., 2008
5	NeuroRD	Stochastic diffusion simulator to model intracellular signaling pathways	XML	http://krasnow1.gmu.edu/CENlab/software.html	SCR_014769	Oliveira et al., 2010
6	Simbiology	MATLAB’s systems biology toolbox (Mathworks)	sbproj	https://www.mathworks.com/products/simbiology.html		Schmidt and Jirstrand, 2006
7	STEPS	Simulation tool for cellular signaling and biochemical pathways to build systems that describe reaction–diffusion of molecules and membrane potential	SBML	http://steps.sourceforge.net/STEPS/default.php	SCR_008742	Hepburn et al., 2012
8	VCell	Simulation tool for deterministic, stochastic, and hybrid deterministic–stochastic models of molecular reactions, diffusion and electrophysiology	SBML, CellML	https://vcell.org/	SCR_007421	Schaff et al., 1997
	*Cellular level*					
9	NEURON	Simulation environment to build and use computational models of neurons and networks of neurons; also subcellular simulations with the reaction–diffusion module	SONATA (after conversion) for networks, but can also use NeuroML and SBML	https://neuron.yale.edu/neuron/	SCR_005393	Carnevale and Hines, 2009; Hines and Carnevale, 1997
	*Network level*					
10	BRIAN	Simulation tool for spiking neural networks	SONATA	https://briansimulator.org/	SCR_002998	Goodman and Brette, 2008; Stimberg et al., 2019
11	NEST	Simulation tools for large-scale biologically realistic neuronal networks	SONATA (after conversion)	https://www.nest-initiative.org/	SCR_002963	Diesmann et al., 1999
12	PyNN	A Common Interface for Neuronal Network Simulators	SONATA	http://neuralensemble.org/PyNN/	SCR_005393	Davison et al., 2008
	*Multiscale*					
13	MOOSE	Multiscale object-oriented simulation environment to simulate subcellular components, neurons, circuits, and large networks.	SBML, NeuroML	https://moose.ncbs.res.in/	SCR_002715	Ray and Bhalla, 2008
14	NetPyNe	Multiscale models for subcellular to large network levels	NeuroML/SONATA	netpyne.org	SCR_014758	Dura-Bernal et al., 2019
15	PottersWheel	Comprehensive modeling framework in MATLAB		https://potterswheel.de/	SCR_021118	Maiwald and Timmer, 2008
16	SYCAMORE	Building, simulation, and analysis of models of biochemical systems	SBML	http://sycamore.h-its.org/sycamore/	SCR_021117	Weidemann et al., 2008
17	The Virtual Brain	Create personalized brain models and simulate multiscale networks	hdf5, Nifti, GIFTI	https://thevirtualbrain.org/	SCR_002249	Sanz-Leon et al., 2015

In simulations, molecular reactions and cellular- or network-level behavior and interactions are often described by a set of ordinary differential equations (Hodgkin and Huxley, 1952) or stochastic differential equations (Manninen et al., 2006). Standardized model specifications like those described above are interpreted by simulators as terms and parameters in the differential equations, and given appropriate initial values, the mathematical equations are solved using numerical solvers. In systems biology, Boolean networks have also been applied successfully, for example Calzone et al., 2010. Another useful modeling paradigm is *rule-based modeling* (Boutillier et al., 2018; Danos and Laneve, 2004; Harris et al., 2016).

For biochemical models, a common approach is to model them deterministically using the law of mass action and Michaelis–Menten kinetics; these models are continuous in state space and macroscopic. This approach is accurate for large numbers of molecules and is more computationally efficient than stochastic approaches, when applicable. In contrast, stochastic approaches are more accurate but numerically costly; however, stochastic methods must be favored at low molecule numbers to give accurate results. Reaction–diffusion simulations, which take into account that particular molecules can diffuse, require additional geometrical information. The geometry can be relatively simple such as the morphology of a reconstructed neuron in a multicompartment model or more intricately detailed such as a tetrahedral mesh. Some reaction–diffusion simulators, such as MOOSE (Ray et al., 2008), NeuroRD (Jedrzejewski-Szmek and Blackwell, 2016), and STEPS (Hepburn et al., 2012), allow both stochastic and deterministic modes of simulation.

Table 3 provides examples of widely used model simulation frameworks for different spatial scales – from the molecular level to networks of spiking neurons and whole brain regions (Brette et al., 2007; Sanz-Leon et al., 2015; Einevoll et al., 2019) It is, of course, valueble when such simulators and also other software are *interoperable*. This is facilitated by standardized, machine readable formats for model descriptions. Interoperability can have another meaning in addition to file format compatibility among simulators and other software: the possibility to run two simulation frameworks in parallel (e.g., at different biological scales), denoted cosimulation, and establish communication between them (Cannon et al., 2007; Djurfeldt et al., 2010; Newton et al., 2018).

### Model refinement

Once a model can be simulated, the next critical step in model development is model refinement: the adjustment of the model structure and parameter values (Figure 2, box 2b). Here, we emphasize *automation* and the use of new *data science methodologies* to improve this process. This is important as the amount of data rapidly increases, and models become more complex. Other important aspects of model refinement are the inclusion of measures of uncertainty into the parameter estimates and predictions, as well as making the refinement process reproducible.

#### Model structure constraints

There are often physical or mathematical conditions that relate to the structure of the model, which must be respected for the model to be valid, in most cases to make sure that physical laws of some kind are not violated. Automatic methods for such structure refinement require machine readable standardized model specifications that can be updated easily in an automatic fashion, like those described above. Such standardized formats also enable *interoperability* so that different software can be used for different types of refinement on the same model.

For models at the biological scale of neurons or circuits, model structure refinement might include checking morphological structures of dendrites or axons for closed paths (Ascoli et al., 2007) or performing unit checking. The use of model description standards can aid these efforts by allowing easy access to model components in an object-oriented way, and at least one library for interacting with description standards provides support for unit checking (Cannon et al., 2014).

Models at the subcellular level often require structure refinements that include modifications due to mass conservation laws and thermodynamic parameter relationships (Wegscheider, 1902; Klipp et al., 2016, chapter 4). Mass conservation means that the total quantity of a substance should not change, for example the total of all forms of protein kinase A (holoenzyme or bound to cAMP) should remain constant. The exception is when molecules are deliberately introduced or removed from the simulation. Thermodynamic constraints require that the combined reaction rates from a set of substrates to a set of products need to be the same regardless of the route taken. Checking for conservation laws and thermodynamic constraints can be done in a semi-automatic fashion through stoichiometric analysis, as is done for conserved substances by the Copasi simulator (Hoops et al., 2006). Software is also available to introduce such relationships semiautomatically into the SBtab format (Santos et al., 2021).

#### Parameter estimation and uncertainty quantification

The refinement of model parameter values, called *parameter estimation* or *calibration*, is typically an iterative process where the model is simulated, compared to experimental data, and then updated — repeatedly (Figure 2, box 2b). This requires model and experimental data in a machine readable (preferably standardized) form and software that efficiently searches the parameter space. Parameter estimation is a field under development, and here we describe classical optimization methods as well as some data science methods for uncertainty quantification more novel to this field. More information is given in, for example, Ashyraliyev et al., 2009; Mitra and Hlavacek, 2019; Sun et al., 2012.

In an ideal world, parameters such as rate constants and species concentrations would be directly reported in publications. The reality is that there are many experiments that potentially provide good constraints for parameters, but do not directly report the parameters in the form needed for a model. For example, the rate of appearance of the phospho-form of a protein depends on the concentration of the phosphorylating enzyme, its kinetics, the concentration of the substrate, and possibly other factors. What is most useful for a model is an estimate of the kinetic rate because enzyme and substrate concentration are determined by other reactions. The kinetic rate, and other parameters, could potentially be inferred (indirectly) from experimental data for quantities like the relative activation of the enzyme or turnover of the protein. The overall approach, denoted parameter estimation, is as follows.

- Select the *experimental data* to be used for the parameter estimation, and formalize a mapping between model output and experimental readouts.
- Describe the prior assumptions made on possible parameter ranges. These assumptions guide your parameter estimation process and could be based on experimental information or physical constraints, for example all parameters are often assumed to be nonnegative.
- Calibrate the model, that is do parameter estimation, either through optimization techniques that provide one good parameter set, or through an approach resulting in a distribution of good parameter values.
- Quantify the uncertainty in your parameter estimates. Ideally by describing the whole parameter space that would correspond to a good fit with the data used for parameter estimation through for example a Bayesian approach, or at least by performing a sensitivity analysis.
- Update the model specification with the new parameter values, and if possible, uncertainty estimates.

In the section ‘Model specification and data’, we describe different formats and practices that can be used to contain all this information, keeping FAIR aspects in mind.

Numerous approaches have been developed for finding parameters that allow a model to match experimental data through optimization (Ashyraliyev et al., 2009; Villaverde et al., 2019). For most models, the optimization method must be able to deal with multiple local minima. A key aspect of optimization is to specify the function that compares simulations with experimental data to find the best set of parameters. This function is often called the objective function, cost function, or energy function and includes a decision on how the different experiments should be weighed against each other. The most common function to use is the sum of the squared differences between simulations and data, weighted by the measurement error (Ashyraliyev et al., 2009). Some existing tools that can be used for parameter estimation are listed in Table 4.

**Table 4.** Tools for model refinement and analysis. This table exemplifies some common and new tools for parameter estimation and different types of model analyses.

	Name	Purpose	Interchange file formats supported	Homepage	RRID	Reference
1	Ajustador	Data-driven parameter estimation for Moose and NeuroRD models. Provides parameter distributions.	CSV, MOOSE, NeuroRD	https://neurord.github.io/ajustador/		Jedrzejewski-Szmek and Blackwell, 2016
2	AMICI	High-level language bindings to CVODE and SBML support.	SBML	https://amici.readthedocs.io/en/latest/index.html		Fröhlich et al., 2021
3	BluePyOpt	Data-driven model parameter optimization.		https://github.com/BlueBrain/BluePyOpt	SCR_014753	Van Geit et al., 2016
4	PottersWheel	Parameter estimation, profile likelihood: determination of identifiabilty and confidence intervals for parameters.	SBML	https://potterswheel.de/	SCR_021118	Maiwald and Timmer, 2008; Raue et al., 2009
5	pyABC	Parameter estimation through Approximate Bayesian Computation (likelihood free Bayesian approch).	PEtab, SBML via AMICI	https://pyabc.readthedocs.io/en/latest/		Klinger et al., 2018
6	Simbiology	MATLAB’s systems biology toolbox (Mathworks), performs, for example, parameter estimation, local and global sensitivity analysis, and more.	SBML	https://se.mathworks.com/products/simbiology.html		Schmidt and Jirstrand, 2006
7	Uncertainpy	Global Sensitivity Analysis	NEURON and NEST models	https://github.com/simetenn/uncertainpy		Tennøe et al., 2018
8	XPPAUT/AUTO	Model analysis including phase plane analyses, stability analysis, vector fields, null clines, and more XPPAUT contains a frontend to AUTO for bifurcation analysis.		http://www.math.pitt.edu/~bard/xpp/xpp.html	SCR_001996	Ermentrout, 2002 (XPPAUT)
9	pyPESTO	Toolbox for parameter estimation	SBML, PEtab	https://github.com/ICB-DCM/pyPESTO/	SCR_016891	Stapor et al., 2018
10	COPASI	Simulation and analysis of biochemical network models	SBML	https://copasi.org	SCR_014260	Hoops et al., 2006
11	PyBioNetFit	Parameterizing biological models	BNGL, SBML, BPSL	https://bionetfit.nau.edu/		Mitra et al., 2019
12	Data2Dynamics	Establishing ODE models based on experimental data	SBML	https://github.com/Data2Dynamics/d2d		Raue et al., 2015
13	HippoUnit	Testing scientific models	HOC language	https://github.com/KaliLab/hippounit		Sáray et al., 2021

Criticisms are often heard about the large numbers of parameters in modeling studies implying that ‘anything can be fitted’. Implicitly, this means that the model lacks explanatory and predictive power. This is a more common criticism for models that include many details, like mechanistic models built at a fine granularity in a bottom-up manner. However, it is important to note that the mechanistic model structure itself puts a lot of restrictions on the possible model behaviors. Also, since the parameters and variables that are associated with mechanistic models can be mapped to biological entities, these are also restricted through known physiological constraints, increasing the specificity of the model. Thus, it can actually be very hard (or impossible) to fit a specific dataset. If solutions exist, however, it is often so that there are many parameter sets that produce simulation results with a good fit to data, thus explaining the data equally well (Gutenkunst et al., 2007). Different parameter sets may however give different model predictions (Eriksson et al., 2019). This calls for methods that go beyond the use of a single parameter set, accounting for uncertainty in the modeling of neuroscience systems. Such uncertainty can come from sparse experimental data or structural unidentifiability (Raue et al., 2009) or it may reflect actual biological variability (Marder et al., 2015). In key studies, Prinz et al., 2004 investigated the possibility of variability in small neural invertebrate circuits using computational models. With an ensemble model approach, they showed in models of the pyloric rhythm of the crustacean stochastic ganglion that similar oscillatory network activity could arise from widely disparate circuit parameters (Prinz et al., 2004), suggesting that there could be considerable animal-to-animal variability in many of the parameters that control network activity. This has also been confirmed in experiments (Schulz et al., 2006).

Although computationally expensive, Bayesian approaches provide the perfect tools for describing such uncertainty in parameter estimates (Vanlier et al., 2013). These approaches often use Markov Chain Monte Carlo (MCMC) methods to explore the possible parameter space, resulting in a joint distribution describing good parameter values (e.g. Girolami and Calderhead, 2011). However, currently, MCMC methods are only applicable for medium sized models, on the order of dozens of parameters. A less costly approach with a similar goal is profile likelihood (Raue et al., 2009), which provides a possible range for each of the uncertain parameters, but not the *joint* parameter distribution. Global sensitivity analysis can also be used to investigate uncertainty, assuming that the parameters have specific predefined distributions (Tennøe et al., 2018).

Parameter space exploration is a process that could benefit from additional automation. For example, many conventional plasticity models are ‘manually tuned’. Nonetheless, parameter optimization has been combined into different workflows, often using custom scripts to interface with an optimization tool. FindSim (Viswan et al., 2018) is a framework for integrating many individual electrophysiological and biochemical experiments with large, multiscale models so that model output can be compared with experiments in an optimization workflow. Santos et al., 2021 describe an automatic workflow for parameter estimation using an SBtab file as input, rendering itself to automatic parameter estimation through many of the optimization tools that exist within Matlab, and with a final step of global sensitivity analysis. Ajustador (Jedrzejewski-Szmek et al., 2018) is an optimization algorithm for models specified in declarative format for either MOOSE or NeuroRD that allows specification of the weights of various data features. SciUnit (Omar et al., 2014) provides a Python-based framework for creating standardized tests that compare model outputs to experimental data. NeuronUnit provides a domain-specific repository of tests for use within the SciUnit framework that neuron models must pass to demonstrate validity when modeling at the detailed cellular level. These tests can also be used as the basis for model optimization (Gerkin et al., 2018). More parameter estimation tools are listed in Table 4.

To be able to *reproduce* the model refinement process, it is important that the model, experimental data, and prior parameter assumptions are well described and explicitly specified, as described above. If this is the case, then ideally different parameter estimation tools should come to similar conclusions, at least if a methodology including a distribution of possible parameter values is used.

#### BCM example

Parameter estimation is not confined to data-driven, mechanistic models. Even abstract models reflect the observables that they set out to explain. Thus, one can ‘tune’ parameters in abstract models to data using the same techniques discussed above. The authors of the original BCM model (Bienenstock et al., 1982) did this to obtain observed ocular dominance properties in their simulations. Another early model of synaptic plasticity (Lisman, 1989) explicitly proposed a set of pathways that could result in bidirectional plasticity equivalent to the BCM curve and implemented chemical reactions with rates that were tuned to obtain properties consistent with the BCM model. Today more efficient parameter estimation methods are used, and a next step could be to quantify the uncertainty in the parameter estimates and predictions through a Baysian methodology, if possible, or perform a global sensitivity analysis, to understand the effects of different parameters on the model behavior.

### Model validation and usage

Following the model refinement process, we obtain the final version of the model that can be validated, analyzed, and used in simulation studies to make predictions and test hypotheses (Figure 2, Box 3). In multiscale modeling the output from the model can also be used as the input for another scale. Ideally, before the model is used for predictions, it is *validated* against data that were not used in the model construction and refinement process. However, data may be sparse requiring that validation occur through experimental tests of model predictions. After the model is finalized, the predictions from the model can be extended and better informed through model analysis. This could be done by *sensitivity analysis*, with tools from *dynamical systems theory* or through *experimental design*. Some examples of tools for model analysis are listed in Table 4.

**Figure 4.**
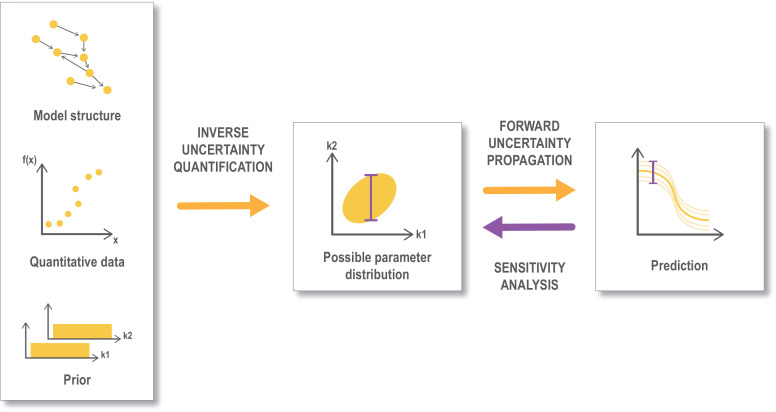
Workflow for uncertainty quantification. An important aspect of data-driven, mechanistic modeling is to describe the uncertainty in the parameter estimates (*inverse uncertainty quantification*), that is to find and describe the parameter space that provides a good fit with the selected data. This is often done through Bayesian methodology starting from a model structure, some quantitative data that can be mapped to the output from the model and prior information on the parameters (like assumed ranges or distribution). The parameter space retrieved from this process is referred to as the posterior parameter distribution. This uncertainty is propagated (*forward uncertainty propagation*) to the predictions that we make from the model, by performing simulations from a sample of parameters representing the possible parameter distribution (corresponding to an ‘ensemble model’). Finally a global *sensitivity analysis* can be performed based on the posterior distribution (Eriksson et al., 2019). Global sensitivity analysis are also often performed in other settings (not shown here) directly on a preassumed parameter distribution. Figure modified from Eriksson et al., 2019.

*Sensitivity analysis* is a methodology that investigates how different *input factors*, such as model parameters or model input, affect the model output (Saltelli et al., 2007; Zi, 2011).The output could be the concentration of the active form of a protein as an example. Sensitivity analysis can be performed through *local* methods, which considers the partial derivatives in the close neighborhood of a point in parameter space, or *global* methods, which investigate a much larger region of the parameter space, often with statistical methods. Local methods are computationally efficient, but can be problematic due to limited range. Global sensitivity analysis on the other hand investigates a larger region, but is computationally costly. One example where a global sensitivity analysis was used is the study by Halnes et al., 2009. There are, however, also coarse-grained global sensitivity analysis methods as well as novel methods that interface coarse-grained global with local sensitivity analyses methods. Several studies have also investigated the sensitivity by a so-called one-at-a-time approach, where parameters are perturbed one at a time, for example Gutierrez-Arenas et al., 2014. Finally, a Bayesian approach for uncertainty quantification can also be combined with global sensitivity analysis (Eriksson et al., 2019), as illustrated in Figure 4. Tennøe et al. have recently developed a software for global sensitivity analysis (Tennøe et al., 2018).

In an *experimental design* process, models can be used to simulate a large number of different scenarios corresponding to different potential experimental setups and in this way be used to design experiments that provide the most amount of new information (Kreutz and Timmer, 2009). Statistical methods have been developed for this, often using Bayesian methodology, see for example Liepe et al., 2013.

Another way of analyzing dynamical models is to use tools from *dynamical systems theory*. Such methods typically examine asymptotic stability, provide phase diagrams, and identify bifurcations in parameter space that identify and explain how major changes in model behavior rely on parameters or input. This approach has, as an example, been used to better understand systems level mechanisms (e.g., how a cell goes from silent to spiking, or how oscillatory phenomena in networks might be controlled [Ermentrout et al., 2019; Keeley et al., 2017]). With regard to subcellular-level phenomena important for plasticity, intracellular calcium release (Li and Rinzel, 1994) as well as switch-like behaviors have been investigated, for example CaMKII and protein kinase M – zeta (PKMζ) (Graupner and Brunel, 2007; Helfer and Shultz, 2018; Pi and Lisman, 2008). For instance, stability analysis shows that CaMKII can be at two stable states at resting intracellular calcium concentration (Graupner and Brunel, 2007), and the system of coupled CaMKII and protein phosphatase A (PP2A) could behave as a tristable system that describes LTP and LTD (Pi and Lisman, 2008). The original BCM study also included analysis with tools from dynamical systems theory to test whether the plasticity rule was stable.

## Toward FAIR modeling workflows

There are many options for assembling the resources and approaches described to create specific modeling workflows that follow the steps outlined in Figure 2. A flexible workflow going through all the steps requires:

1. Much effort toward identifying the scientific questions to be addressed and the model prerequisites (see ‘Model foundation’).
2. A well-defined format for the model, the quantitative experimental data, and the assumptions on prior parameter ranges or distributions (see ‘Specifications of model and data’).
3. A simulator that supports a machine readable, standardized specification of the model (see ‘Mathematical formulation and simulation’).
4. A tool for searching the parameter space, that compares the simulations with the experimental data guided by the parameter prior information (see ‘Model refinement’).
5. Tools for model analysis (see ‘Model usage and validation’).
6. Parsing tools to go between the specification of model and data format and internal formats for the model refinement and model analysis tools.

**Figure 5.**
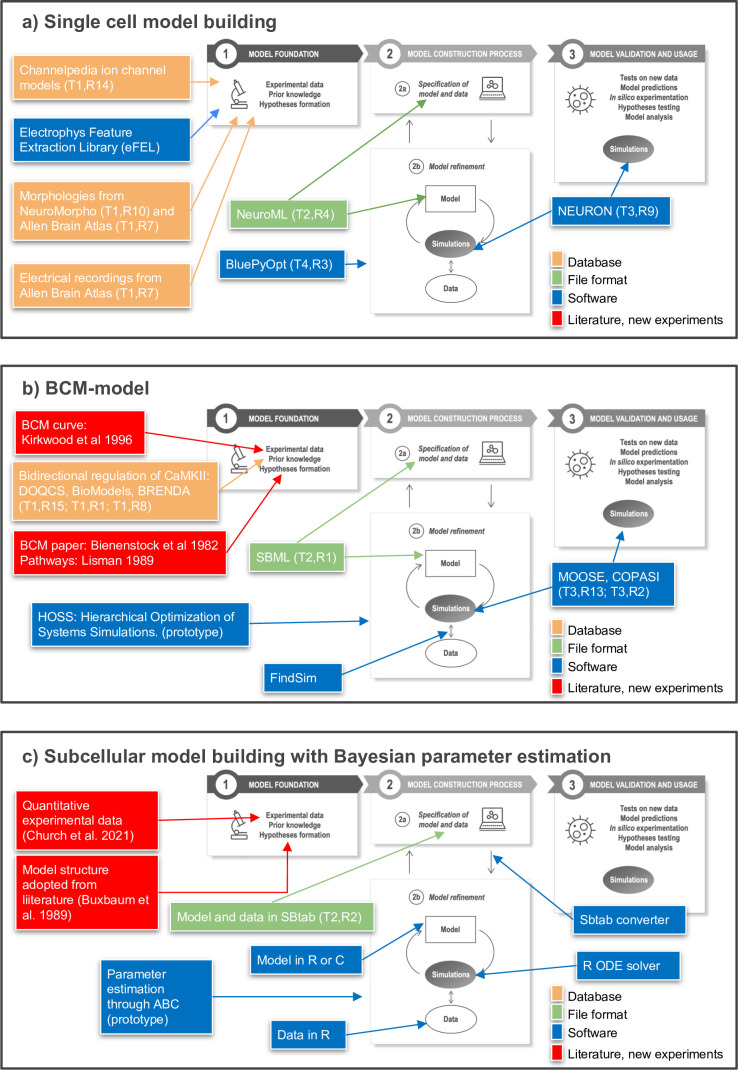
Toward FAIR (Findable, Accessible, Interoperable, Reusable) workflows in neuroscience. Three examples of workflows at different biological scales. Several of the workflow components can be found in the tables of the article as indicated by T (Table) and R (Row) in the figure, for example T1,R4. (**a**) To build a single neuron multicompartmental electrical model, morphologies from databases such as NeuroMorpho or Allen Brain Atlas (http://celltypes.brain-map.org/) can be used (Ascoli et al., 2007; Gouwens et al., 2020). Electrical recordings from Allen Brain Atlas are also available. Features from experimental traces can be extracted using eFEL (https://github.com/BlueBrain/eFEL), and kinetic parameters for ion channel models obtained from Channelpedia. During the neuron model reconstruction step, the model is represented in NeuroML and model parameters such as conductance density are optimized with BluePyOpt. In silico experiments can be performed using NEURON. (**b**) Example workflow for building a chemical kinetic model to implement the Bienenstock–Cooper–Munro (BCM) curve. The expected shape of the curve is obtained from the classic Bienenstock et al., 1982 study, and a first pass of likely chemical pathways from Lisman, 1989. Detailed chemistry and parameters are from databases that cover models (DOQCS, BioModels) and from BRENDA, which hosts enzyme kinetics. Both model databases support SBML, which can be used to define the model and parameters. Several simulators including MOOSE and COPASI can run the SBML model during the optimization step and subsequently for model predictions. For optimization, the FindSim framework (Viswan et al., 2018) compares model outcome to experiments. The score from these comparisons is used by HOSS (Hierarchical Optimization of Systems Simulations https://github.com/upibhalla/HOSS) to carry out parameter fitting. (**c**) An example of a workflow used in subcellular model building with Bayesian parameter estimation. A prototype of the workflow was used in Church et al., 2021, where the experimental data is described. The model structure was adopted from Buxbaum and Dudai, 1989. A smaller demo version can be found at https://github.com/icpm-kth/uqsa (copy archived at https://doi.org/10.5281/zenodo.6625529). Experimental data and model structure are taken from literature and saved in the SBtab format. Scripts written in R convert the SBtab file to R code and the Bayesian parameter estimation is performed with the UQSA software written in R (https://github.com/icpm-kth/uqsa, copy archived at https://doi.org/10.5281/zenodo.6625529). The SBtab files can subsequently be updated with the refined model with new parameter estimates including uncertainties.

In Figure 5, we provide three specific example workflows for subcellular- and cellular-level modeling that traverse these steps in different ways. These examples illustrate recent steps toward FAIR workflows and provide a starting point for further discussion within the community.

Aside from single steps and components, we will now consider some questions that are important for *all* parts of the workflow, and in relation to this we also recommend the article by Goble et al., 2020, which describes FAIR computational workflows from a more general perspective. The first question concerns findability and here we emphasize the importance of metadata and providing unique and stable identifiers to the workflow components. Concerning models and data, some databases provide a unique Digital Object Identifier (DOI) for its constituents. This can be retrieved when the model or data are deposited in the database and included in the metadata of the workflow. Each resource and software used in the workflow should also be indicated using the associated, unique SciCrunch Research Resource Identifier (RRID) (Vasilevsky et al., 2013; Bandrowski and Martone, 2016), if it exists. It can be noted that in order to get an identifier to a more complex research object a general database like Zenodo (European Organization For Nuclear Research, 2013) could be used. As an example it is possible to use Zenodo to get a DOI for a GitHub repository release.

The second question concerns approaches for the packaging, description, and dissemination of the *entire* model building workflow, which is important for transparency and reproducibility. FAIRDOMhub (Wolstencroft et al., 2017) is a repository and collaboration environment for publishing FAIR data, operating procedures, and models for the systems biology community. EBRAINS has a similar ambition to provide a collaboration hub for brain research. Virtual machines or containers (Merkel, 2014) can play a role in reproducing all steps and results of a workflow. Easily accessible technologies such as Jupyter notebooks (Kluyver et al., 2016) can aid in interoperability, dissemination, and collaboration. The Common Workflow Language allows users to fully specify inputs, outputs, and other execution details needed for tools in a workflow and instructions for executing them within containers (Piccolo et al., 2021). The workflow around a model can also be made public analogous to publishing the protocol of a lab experiment, for example via protocols.io (Teytelman and Stoliartchouk, 2015). The recently published Research Object Crate (RO-Crate) provides a way to package and aggregate research ‘artifacts’ with their metadata and relationships (Soiland-Reyes et al., 2022), like spreadsheets, code, examples, and figures.

Finally, we illustrate a FAIR workflow by considering how one would today develop simulations for the BCM curve in Figure 3. Even though the original BCM model and many subsequent experimental studies predate the advent of FAIR principles, it is interesting to think about what would need to be done in order to make them FAIR compliant. Ideally, these studies would be *Findable* through PubMed and other resources. This implies that both metadata and data are easy to locate for both humans and computer programs. We would probably rely more heavily on later models, for example, Hayer and Bhalla, 2005, that are easily *Accessible* and in an *Interoperable* format such as SBML or SBtab. The Hayer model is available via the DOQCS database (Sivakumaran et al., 2003). In order to be *Reusable*, models should be replicable and compatible in different contexts. Quantitative experimental data could today be specified using FindSim or SBtab formats. Both formats support the comparison of model behavior with the experimental data, which is necessary for model refinement. For the model refinement to be reproducible it is important that the prior assumptions on parameters are described. It should be noted that the provenance for the Hayer model is not machine readable, though ideally it would be. Furthermore, more comprehensive metadata and appropriate license information should be included. Finally, since the refined model is defined in SBML, it could be used on any of several dozen simulator platforms (Table 3), and uploaded back to the *Accessible* databases to become part of the FAIR ecosystem.

## Conclusions and outlook

We have investigated how data- and hypothesis-driven modeling approaches can be combined, through a FAIR infrastructure, in order to improve modeling capabilities within the neuroscience community. We suggest a minimal format for the ‘specification of model and data’ (Figure 2, box 2a) in order for the modeling process to be reproducible. We also describe different databases, formats, and software (Tables 1–4) and methods for combining them to achieve FAIR modeling workflows. We believe that such workflows would increase the capacity for combining different types of models, extending models as new data accumulates, and validating existing models.

When it comes to model refinement (Figure 2, box 2b), we have emphasized the importance of uncertainty quantification with for example Bayesian methods (Figure 4) in order to describe the uncertainty in the parameter estimates and model predictions. However, currently such methods are only possible (in practice) for models of the order of some dozens of parameters, pointing to a need for further method development. Concerning approaches for model analysis (Figure 2, boxes 2b and 3) an important aspect is to characterize models more objectively, thus avoiding the situation that the model has been ‘tuned’ to give a certain outcome. This is especially important as the goal of a model often is to reproduce specific observed behaviors in order to validate a hypothesis, rather than being predictive. This is particularly true for abstract, phenomenological models; however, data-driven mechanistic models that are portrayed as predictive often play the same, useful postdictive role. Many studies of such models now conduct parameter sensitivity analysis and parameter explorations to discover regimes in which new properties emerge, thereby providing an unbiased overview of the possible properties of the model.

In the future, it is likely that machine learning tools increasingly will be integrated with traditional modeling approaches as a complement but also a necessity for dealing with high throughput data of varying fidelity (Alber et al., 2019). While machine learning approaches are useful for finding correlations in big data, traditional mechanistic models are important for revealing causal relationships and mechanistic explanations. Mechanistic models also have the added possibility to generalize to new situations not considered during the model construction process.

If we want to understand the brain — from molecules to behavior — we need to model the brain at all biological scales to integrate and interpret various heterogeneous experimental data across scales. Here we have examined the opportunities associated with models arising at the scale where subcellular signaling processes meet the cellular level, corresponding to the field where the systems biology community meets the computational neuroscience community. Although the development of resources that integrate the FAIR principles into the computational neuroscience field has progressed over the last decade (e.g., compare De Schutter, 2008), standards and best practices still need to be further developed and aligned, an important endeavor coordinated by INCF (Abrams et al., 2021). The adoption of FAIR principles is timely due to the recent debut of brain initiatives around the world (Grillner et al., 2016), including the establishment of the International Brain Initiative (International Brain Initiative, 2020). Such efforts will help promote data and model exchange and reuse through international research and infrastructure collaborations.
